# Methodology to derive preference for health screening programmes using discrete choice experiments: a scoping review

**DOI:** 10.1186/s12913-022-08464-7

**Published:** 2022-08-24

**Authors:** David Brain, Amarzaya Jadambaa, Sanjeewa Kularatna

**Affiliations:** Australian Centre for Health Services Innovation, Centre for Healthcare Transformation, School of Public Health and Social Work, Faculty of Health, Queensland University of Technology, Kelvin Grove, 4059 QLD Australia

**Keywords:** Preferences, Discrete choice experiment, Community screening program, Chronic disease

## Abstract

**Background:**

While involving users in healthcare decision-making has become increasingly common and important, there is a lack of knowledge about how to best design community-based health screening programs. Reviews of methods that incorporate discrete choice experiments (DCEs) are scarce, particularly for non-cancer illnesses like cardiovascular disease, diabetes and liver disease. We provide an overview of currently available applications and methods available by using DCEs in health screening programs, for chronic conditions.

**Methods:**

A scoping review was undertaken, where four electronic databases were searched for key terms to identify eligible DCE studies related to community health screening. We included studies that met a pre-determined criteria, including being published between 2011 and 2021, in English and reported findings on human participants. Data were systematically extracted, tabulated, and summarised in a narrative review.

**Results:**

A total of 27 studies that used a DCE to elicit preferences for cancer (*n* = 26) and cardiovascular disease screening (*n* = 1) programmes were included in the final analysis. All studies were assessed for quality, against a list of 13 criteria, with the median score being 9/13 (range 5–12). Across the 27 studies, the majority (80%) had the same overall scores. Two-thirds of included studies reported a sample size calculation, approximately half (13/27) administered the survey completely online and over 75% used the general public as the participating population.

**Conclusion:**

Our review has led to highlighting several areas of current practice that can be improved, particularly greater use of sample size calculations, increased use of qualitative methods, better explanation of the chosen experimental design including how choice sets are generated, and methods for analysis.

**Supplementary Information:**

The online version contains supplementary material available at 10.1186/s12913-022-08464-7.

## Background

In recent years, there have been increased calls for user’s involvement in healthcare decision-making [1, 2]. User involvement can support decision-making at the planning and rolling out stages and these views can be elicited quantitatively, qualitatively or using a mixed-methods approach [3, 4]. Discrete choice experiments (DCEs) are a quantitative technique for appraising user choices, and are increasingly used to inform decisions on healthcare treatments, diagnostics and screening programs [5].

Since 1990, there has been a marked increase in years lived with disability from non-communicable diseases including cancer, cardiovascular and liver disease [6]. Over the next 15 years, the human and economic costs of these diseases are estimated to total more than US$7 trillion in developing countries alone [7]. To address this, preventive interventions and early detection of disease is a key area of research. A comprehensive approach, which includes health screening programs, are often being recommended [7, 8]. Professional societies recommend health screening programs, because patients with screen detected chronic disease are more likely to have early-stage disease and better prognosis [9, 10]. For example, patients at higher risk for cancer or fibrosis due to chronic hepatitis B, cirrhosis, and non-alcoholic fatty live disease are recommended to have surveillance examinations such as imaging and histology tests [11, 12]. Despite the many available screening programs and current recommendations, health screening uptake remains low in many countries [13, 14]. To increase uptake there is a need to quantify users’ preferences for health screening programs, using the robust method of discrete choice experiments [3].

Studies reporting DCE methods and results can provide decision-makers and researchers with valuable insights how health screening programs could be delivered more effectively and more efficiently [15]. In a DCE, participants state their preferred option from a set of alternative services, described by the same attributes but perhaps differing in their amount, known as levels [3].

The use of DCEs in the healthcare setting is increasing but reviews of methods that incorporate DCEs are scarce. Systematic reviews of health related DCEs have increased from seven studies in 1999 [16], to 30 studies in 2008 [17] and 98 studies in 2015 [18]. These reviews included and analysed 129, 114 and 301 DCEs respectively, but did not categorise by treatment, screening, or prevention options. The reviews identified a substantial variation [17] as well as inadequate reporting of the methods [18], inhibiting quality assessment. Therefore, there is need to summarize the methods used in previous DCE studies. There have been some reviews of the DCEs regarding health screening programs, including cancer screening [19], colorectal cancer screening [20, 21], and newborn screening [22]. Generally, these reviews concluded that the specific methodological issues raised when conducting DCEs in health screening programs, indicating the methodology needs to be evolved. To our knowledge, no review has summarised studies that have elicited preferences for chronic disease screening programmes such as cancer screening programs as well as cardiovascular or liver disease screening programs besides increased number of screening programmes for chronic conditions [13, 14].

This study was designed to conduct a scoping review of DCEs of health screening programs for chronic conditions including cancer, cardiovascular and liver disease screening programs. The objectives were to: (1) identify published studies using DCEs in chronic condition screening programs and synthesize current methodology used in previous DCE studies; (2) assess the quality of included DCE studies, and (3) provide recommendations for future design of DCEs in relation to community-based health screening programs as well as an item list for developing attributes of DCEs in community screening.

## Methods

A scoping review was considered the most appropriate study design [23] to identify the methodologies used in DCEs in health screening programs, describe the key characteristics of DCEs used, and identify key gaps in this research field. This study followed the Preferred Reporting Items for Systematic Reviews and Meta-Analyses extension for Scoping Reviews (PRISMA-ScR) statement for processing and reporting scoping reviews [24, 25] (Appendix 1). A review protocol was developed with search methods and inclusion criteria specified in advance (Appendix 2). As this is a scoping review, registration in PROSPERO was not applicable.

### Inclusion and exclusion criteria

This scoping review included studies meeting the following criteria: (1) applied a discrete choice experiment method; (2) was used to elicit consumer/provider/stakeholders’ preferences towards a chronic disease screening program; and (3) published in English since January 2011. The target population of this review is consumers, healthcare providers and stakeholders who were involved in a chronic disease screening program. Studies were excluded if the DCE:

- was a non-community-based screening program (intended to focus on population/community-based screening programs only).
- was associated with prenatal screening, newborn screening, communicable disease screening, or genetic testing (as the review of the DCEs regarding newborn screening [22] was conducted recently and the current review intended to focus on non-communicable disease screening programs such as cancer screening as well as liver and cardiovascular disease screening programs).
- was a scholarly review, letter to the editor, commentary, news article or conference abstract.

Final decisions regarding the inclusion or exclusion of studies were made by consensus among all reviewers (SK, DB and AJ). Full inclusion and exclusion criteria for selection of studies are shown in the protocol in Appendix 2.

### Search strategy

Four electronic databases (EMBASE, MEDLINE, PubMed, National Health Service Economic Evaluation Databases from 01 Jan 2011 up to 04 Mar 2021) were searched with the assistance of a librarian to identify the studies that applied a DCE method to elicit consumers’, providers’ or stakeholders’ preferences towards a health screening program. The following search terms were used: “discrete choice*” or “stated preference*” or “conjoint analysis*” and “consumer*” or “patient*” or “health care personnel*” or “stakeholder*” and “preference*” or “value*” and “screening” or “surveillance” or “health assessment”. The search was restricted to the English language and by publication since 2011. The reference lists of included studies were manually searched for other relevant studies.

### Data extraction and quality assessment

After removal of duplicates using EndNote software, studies were imported into a web-based review program – Rayyan [26]. The titles and abstracts were screened for relevance and adherence to eligibility criteria by three reviewers, independently, and data were extracted using a predesigned extraction form. In preparation for data extraction, a predesigned data extraction spreadsheet was piloted and iteratively revised by the research team. The author (AJ) extracted data using the final version of this spreadsheet in Microsoft Excel. The data extraction sheet was pilot tested on five studies and was revised to include:

- background information - data source, period of publication, location, sample size, age group;
- development of choice set - methods for attributes and levels selection, number of attributes, levels, alternatives with or without opt-out option;
- attribute development - design type, plan, software;
- econometric analyses - econometric analysis model, software;
- presented outcome measures (Appendix 3).

We followed a five-step development process for data extraction and analysis of choice sets [27]. This is a rigorous and systematic approach to DCE development which is useful to establish methods for reducing and prioritising attributes. Additionally, more general attribute domains: process attributes, outcome attributes, cost attributes, and others - proposed by existing systematic reviews [28] were used to extract and analyse data as the current scoping review aimed to summarise the methods used in DCEs in community health screening programs.

The quality of each study was assessed using a list of 13 criteria, which covers all four key stages of a discrete choice experiment, using a previously published and validated approach [29]. Each item in the checklist was scored as having ‘met the criteria in full’ (‘1’) and ‘partially met or did not meet the criteria’ (‘0’). Overall compliance with the checklist was calculated as the proportion of the checklist criteria addressed by the study. The quality assessment for each study is presented in Appendix 4.

## Results

### Search strategy

**Fig. 1:**
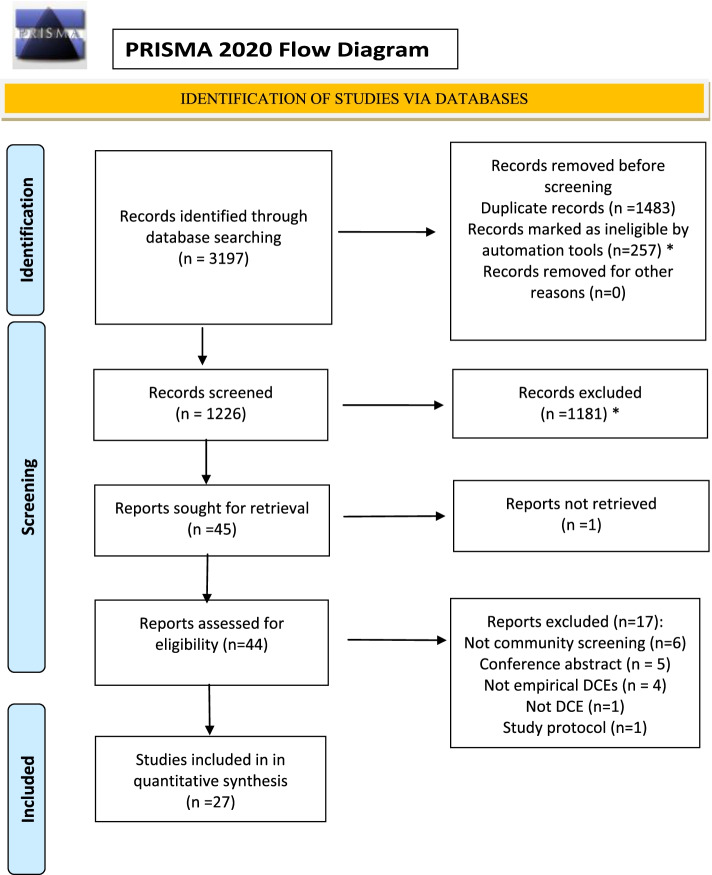
PRISMA flow diagram showing process of study selection for inclusion in review

Figure 1 details the flow of studies through the review process. A total of 3197 articles were identified in the electronic database search, of which 1483 were duplicates. Titles and abstracts for 1226 unduplicated abstracts were reviewed by three authors (SK, DB, and AJ) using Rayyan [26], with a further 1184 articles excluded. Disagreements (*n* = 12) were resolved by consensus among authors. One study was not able to be retrieved. Of the 44 studies assessed for eligibility, 27 fully satisfied the pre-determined inclusion criteria.

### Quality assessment

All studies were scored against a list of 13 criteria [29], with median count of 9/13, and a range between 5 [30] and 12 [31]. Across the 27 studies, majority of studies (80%) had the same overall score and response to individual criteria except a few studies that scored between 5 and 6.

### Background information of included studies

Table 1 provides an overview of descriptive information for the 27 included studies. Only one of 27 studies examined preferences for a cardiovascular disease screening program [32], while the remaining studies investigated preferences for cancer screening programs. The most common objective was to explore the general population’s preferences for a health screening program, which was examined in 21 studies. A median of 685 participants were approached with considerable variation among the studies (range 46 to 4000). When a general population was used, the median remained the same. There was no discernible increase in the number of participants approached when choice sets with higher number of attributes and levels were used. Of 27 DCEs, three had sample sizes less than 100 respondents, whereas eleven of the 27 DCEs had a sample size greater than 1,000 respondents. Only 18 studies reported sample size considerations such as parametric approach [33], Orme’s rule-of-thumb [31, 34–39], and referring studies [40, 41].

The methods of data collection varied between studies but were mostly either self-completed online choice sets or self-completed paper-based choice sets. Participants in 13 of 16 online administered surveys were recruited via an online survey panel. Response rates varied from 5 to 6% [38, 42] to 100% [34, 43, 44]. High response rates were reported in studies where participants were directly invited and data were collected via face-to-face interview [34, 45]. Low response rates were reported in studies where participants were invited via email or traditional mail [36, 38]. In studies where data were collected through an online survey panel, there was a large variation in response rates (6-100%) [42, 44]. Several studies offered a reimbursement to participants.

**Table 1:** DCE background information on the 27 included studies

Item	Specification	Number of studies
Country of origin	Europe	16
	US	5
	Australia	4
	Asia	2
Screening program of interest	Cancer screening program^a^	26
	Cardiovascular disease screening program	1
Target population	General public	21
	Healthcare provider	5
	Healthcare provider and stakeholder	1
Main objective	Consumer preference	21
	Health professionals’ preferences	6
Sample size calculation reported	Yes	18
	No	9
Ethical approval	Approved by international organisation	2
	Approved by local organisation	16
	Not required	1
	Not reported	8
Administration of survey	Self-completed (online)	13
	Self-completed (paper based)	7
	Self-completed (paper based/online)	3
	Face to face interview	4
How participants were recruited	Via an online survey panel	12
	Via an online survey panel/snowball	1
	Via email	1
	Via mail	4
	Via email/mail	2
	Directly invited	5
	Not clearly reported	2
Reimbursements	Money	8
	Gift	1
	Voucher	1
	Not reported	17

^a^Colorectal, breast, cervical, prostate, oesophageal, lung, skin cancer screening programmes

### Developing choice sets

The first step in developing choice sets is to generate a list of attributes using information from literature reviews and qualitative work. Twenty-two of 27 studies conducted literature and/or systematic reviews, and the remainder used: a small qualitative study [42], semi-structured interviews with the target population and healthcare providers [35], or existing research [36, 40, 43]. Ten studies identified attributes and levels through a combination of literature reviews and qualitative work with either a specified target population or identified experts. Interviews were conducted in nine studies, with the majority conducting interviews with both experts and a specified target population, with variable number of participants. For example, De Bekker-Grob, Rose [46] completed interviews with both experts in the field of prostate cancer screening (*n* = 8) and men aged 55–75 years (*n* = 8) while Li, Liu [45] interviewed rural women aged 30–65 (*n* = 15) and clinical experts in the field of cervical cancer screening, to get feedback on the list of attributes created from a literature review. Six studies used focus group: two groups with four participants each [31], eight focus groups with eight women each [34], three focus groups with seven participants each [47], and one focus group with eight individuals [41]. Two of these six did not state how many participants were involved in the focus group [42, 48].

In the second step, a ‘long list’ of attributes was screened by experts to determine whether they would be feasible or meaningful to include in a DCE study. Nine studies involved experts’ opinion in selecting attributes and levels of attributes. Where attributes and levels were created from literature review only (*n* = 3), literature review combined with qualitative work (*n* = 3), or literature review combined with existing research or data (*n* = 3) were validated with expert opinion, according to the area of research.

In the third step, a structured prioritisation or ranking exercise was used. Ten studies reported that interviewers or focus group participants were asked to rank the attributes from most important to least important, with respect to their preferences for screening [33, 40–42, 44, 46, 47, 49–51] to identify the relative importance of various attributes. Following this ranking exercise, with or without subsequent panel discussion, a set of exclusion and inclusion criteria was agreed with reference to a study objective.

In the fourth step, qualitative and/or quantitative pilot tests were undertaken to validate the scenarios and content, face validity, usability, robustness, and likely response rate to DCE questionnaires. Two-thirds of studies reported piloting their surveys before full rollout. There was great variation in piloting, ranging from a qualitative face-to-face interview with only nine individuals from the target population [40], to a two-step procedure including face-to-face interview and online questionnaire completed by 116 participants [50]. Two studies used “think aloud” techniques to check for any problems in interpretation of the attributes, levels, and face validity [47, 51]. In one study [41] the participants were asked to verbalize their thought process during decision-making. There are two types of think aloud: (1) *concurrent think aloud* is where people are asked to verbalise what they are thinking as they complete a certain task, and (2) *retrospective think aloud* asks people to describe what they were thinking after the task has been completed. The study [41] applied a mixed approach: the respondents were asked to think-aloud for two or three choices, and then to reflect after this. If the respondent was silent for a period of time, they were reminded to keep thinking aloud. In the final stage, issues raised from pilot testing were considered and the final attributes and levels were agreed via expert panel discussions. Seven studies stated that no alterations were made after pilot testing [36, 39, 42, 46, 47, 50, 52].

In summary, only a single study applied this five-stage development process and established twelve unlabelled choices of two alternatives, with 5 attributes and 4 levels for each attribute [41]. In this single study, instead of using the “think aloud” technique, researchers conducted two face-to-face pilot studies to ascertain if respondents could manage the length of the questionnaire and to examine its intelligibility, acceptability, and validity [41].

The number of attributes per alternative in the included DCEs ranged from three to ten, with an average of six (Table 2). The most frequently used attributes in these DCEs were details for screening procedure, such as travel time, location, interval, false positive/negative test, sensitivity, and specificity (*n* = 26), regarding costs - out of pocket costs, cost to health service, and cost of follow-up care (*n* = 18). Less frequently used outcome attributes were related to health outcomes, such as reduction in mortality, risk of overtreatment, and surgical outcomes (*n* = 14), and others such as scientific evidence, target population, and stakeholder’s action (*n* = 8).

The average number of levels for each attribute was three, with a maximum of six and a minimum of two. Ten studies included 5 attributes, followed by six studies reporting 6 and other six reporting 7 or more attributes. Sixteen studies used generic or unlabelled scenarios; six studies used labelled scenarios while four did not report their approach clearly enough to tell. A single study used both labelled and generic scenarios [53]. The majority of included studies included two alternatives (*n* = 14), not including an opt-out option, with four studies including three alternatives. Almost half of the reviewed studies (*n* = 12) did not include an opt-out option.

**Table 2:** Developing choice sets

Item	Specification	Number of studies
Methods for attributes and levels selection	Literature review, expert’s opinion	3
	Literature review, expert’s opinion, qualitative work^a^	3
	Literature review, expert’s opinion, existing data	2
	Literature review, expert’s opinion, existing research	1
	Literature review, qualitative work^a^	10
	Literature review, existing research	2
	Literature review, existing data	1
	Qualitative work only	2
	Based on existing research	3
Pilot testing	Yes	18
	No	1
	Not reported	8
Number of attributes	3	1
	4	4
	5	10
	6	6
	7 or more	6
	Average number of attributes	5 to 6
Attributes covered^e^	Procedure attributes	26
	Cost attributes	18
	Outcome attributes	14
	Other	7
Number of levels	2 to 3	13^b^
	2 to 4	6^c^
	2 to 5	5^d^
	3 to 4	2
	4 to 6	1
Labelling	Generic	16
	Labelled	6
	Both	1
	Not clearly reported	4
Number of alternatives (not including opt-out option)	1	2
	2	20
	3	4
	4 or more	1
Opt-out option	Yes	15
	No	12

^a^Qualitative work: Interview with experts and/or stakeholders and/or healthcare providers and/or target population; and/or focus group; and or secondary analysis of qualitative data; ^b^ Includes 2 and 3; ^c^ includes 4; ^d^ includes 5; ^e^ Total more than 27 as each study may use many attributes

### Generating experimental design

Table 3 shows the current trends with respect to experimental design and construction of choice sets. Twenty-five studies used a fractional factorial design; the remaining two studies used a full factorial design. Of the 25 studies that used a fractional factorial design, majority (*n* = 16) did not state the type, others used orthogonal (*n* = 6) or balanced (*n* = 2) or both (*n* = 1) types. The most common measure to select the design type was D-efficient (*n* = 16). Only one study used C-efficient, and the rest ten studies did not state this. Seventeen studies used only a main effects design, while five studies used a main effect plus two-way interaction design [33, 36, 47, 49]. Five studies did not report this aspect of the design plan.

Eleven studies reported using blocks when generating the experimental design (Table 3). On average, studies with blocked design had 650 participants, each of whom completed 10 choice sets, whereas studies without blocking had an average of 230 participants, completing 20 choice sets. Norman, Moorin [43] created an unblocked design with 100 unique choice sets and included those participants in the analysis who completed at least one choice set.

**Table 3:** Experimental design of included studies

Design aspect	Specification	Number of studies
Design type	Fractional factorial	25
	Full factorial	2
Fractional factorial type	Orthogonal	6
	Balanced	2
	Both	1
	NA	2
	Not stated	16
Design plan	Main effects plus two-way interactions	5
	Main effects only	2
	Not clearly reported but main effects only in analysis	15
	Not reported and unclear from analysis	5
Measures to select design	D-efficiency	16
	C-efficiency	1
	Not stated	10
Design software	Ngene	8
	SAS	6
	Sawtooth	5
	SPSS	2
	Not specified	6
Blocking	Yes	11
	No	14
	Not sure/unclear	2
Number of choices per respondent	1–5	1
	6–10	5
	10–15	13
	16–20	6
	21 or more	2

### Conducting econometric analyses

There was considerable variation in methods of econometric analyses between the reviewed studies (Table 4). Ten studies analysed their preference data using modelling techniques such as mixed logit (*n* = 6) and Hierarchical Bayes mixed logit (HB-MXL) model (*n* = 4). Multinominal logit (MNL) and Latent Class logit were used each in three studies while Mixed Multinominal logit (MMNL) and logit were used in two studies. Few studies employed two or more models [42, 43, 47, 48].

Several authors explained why they chose a certain model. For example, de Bekker-Grob, Donkers [47] explained that given their interest in accounting for systematic preference heterogeneity and also taking scale effects into account (i.e. how consistent individuals make their choices), a Heteroscedastic model within a four step approach was employed to determine optimal utility function. Others used Latent class logit model with explanation of appropriateness for identifying different utility functions across different or unobserved subgroups [46, 51]. Another study did not clearly report which model or method was used to analyse weights relating to the utility importance [44].

### Software used

In designing, the most common software packages used to generate experimental designs were Ngene (8 studies) and SAS (6 studies) followed by Sawtooth (5 studies) and SPSS (2 studies). The remaining 6 studies did not clearly indicate the software.

In analysis, six of 27 studies used STATA and five studies used NLOGIT (Econometric Software). Sawtooth and SPSS were each used by three studies while R and SAS each by two studies. Five studies did not report what software they used for analysis.

**Table 4:** Econometric analyses of included studies

Analytic aspect	Specification	Number of studies
Econometric analysis model	Mixed logit/random parameter logit	6
	Hierarchical Bayes mixed logit HB-MXL	4
	Multinominal logit MNL	3
	Latent class logit	3
	Mixed Multinominal logit MMNL	1
	Logit	2
	Random effect probit	1
	Random parameter logit	1
	Generalised multinomial - GMNL	1
	Four models: MNL, Heteroskedastic multinomial – HMNL, HMNL + systematic preference heterogeneity, HMNL + systematic preference heterogeneity + random opt out utility	1
	Two models: MNL/conditional logit, Multinomial probit MNP	1
	Two models: MNL/conditional logit, mixed logit	1
	Two models: MNL/conditional logit, Latent class logit	1
	Not clearly reported	1
Software for econometric analysis	STATA	6
	Nlogit	5
	Sawtooth	3
	SPSS	3
	R	2
	SAS	2
	Pythonbiogeme	1
	Not specified	5

### Outcomes

Twenty-two studies presented their outcomes as utility scores (Table 5). Willingness to pay (WTP) as well as willingness to accept (WTA) were estimated in eleven studies. Only three studies used relative importance and ranking as a primary outcome [35, 44, 54], while another three studies calculated changes to uptake rate according to change in attributes/levels [34, 41, 45]. The remaining three studies presented their results as “other” outcomes, including relative risk [30], choice shares [55], and maximum acceptable risk [38].

There are several function measures that can describe a model’s power, and how well a statistical model fits a set of observations. Thirteen studies reported that they employed likelihood function measures with or without Pseudo R^2^, Bayesian information criterion (BIC), and Akaike information (AIC) criterion to show the model fit. Several studies reported that selection of model was based on goodness of fit measures [42, 43, 47, 54]. Rest of the studies did not report this information.

**Table 5:** Presented outcome measures of included studies

Item	Specification	Number of studies
Presented outcome measure^a^	Utility score	**22**
	Willingness to pay	**9**
	Willingness to accept	**3**
	Relative importance and ranking	**3**
	Uptake rates change according to change in attributes/levels	**3**
	Part-worth utility score	**2**
	Relative risk	**1**
	Choice shares	**1**
	Maximum acceptable risk	**1**

^a^ Total more than 27 as two or more outcomes were presented in one study

## Discussion

 This scoping review gives an overview of available DCE studies evaluating preferences and identified current available applications and key methods used in DCEs to elicit preferences for community health screening programs. Of the 27 included studies, none were related to liver disease which indicates that there is a lack of reporting DCE methods in relation to community screening programmes unless they are related to cancer disease.

Methods in developing choice sets.

Advice regarding methods to develop the attributes and levels of attributes for DCE choice sets are scarce. De Brún, Flynn [27] has given a five-stage development strategy consisting of exploratory work, expert panel discussion, prioritisation, pilot testing and a second expert panel discussion [27]. Moreover, Trapero-Bertran, Rodríguez-Martín [56] recommended to use more in-depth methods such as interviews, sub-group analysis, and expert opinion. These qualitative methods enable sensitive subjects to be discussed and attributes captured, reducing the potential for misspecification of attributes through over-reliance on the views of limited number of experts or researchers. Hence, our review followed a five-step development process proposed by De Brún, Flynn [27] that allows to check whether attributes were developed through qualitative methods. We found that less than 50% of reviewed studies used qualitative methods in selecting attributes and levels of attributes, revealing an area for significant improvement. It is advisable to use qualitative methods to develop and select attributes for DCEs of community screening programs, with clear reporting criteria. Also, we recommend a revised, shortened version of De Brún, Flynn [27] methods for developing attributes for community screening programmes, to facilitate efficiency by excluding the pilot testing stage. This four-stage method could include (1) literature review to develop an initial attribute list, (2) a focus group discussion including patients, clinicians, primary health workers and other stakeholders to revise, to add and remove from the list provided by the review, (3) prioritisation exercise using a sample of clinicians and patients, and (4) expert panel discussion to finalise the attributes and the levels of attributes. After finalising the attributes and the levels of attributes, pilot testing survey will be conducted to ascertain comprehension and understanding of the DCE choice tasks, attributes, and their levels via conducting one-on-one interviews and/or an online survey. The current review allows us to develop a list of items that could be useful in developing attributes of any community screening programme (Table 1, Appendix 5).

### Experimental design

An experimental design is a sample from all possible combinations of the developed attributes and levels. Since a complete combination of all attributes and levels (a full factorial design) is too large to be used in practice, a fractional factorial is frequently used. Lancsar and Louviere [3] recommended to use optimal designs in DCEs in healthcare service because it makes the DCE more robust. Our review found that fractional factorial design is the most popular method to create choice sets using D-efficient as a measure to select designs. Ngene is the most popular software tool for generating experimental designs in DCEs for community-based health screening programmes. Main effects are dominant in the reviewed DCE studies but researchers recommended avoiding small fractional designs that only allows estimation of main effects [3]. Moreover, at a minimum, designs that allow independent estimation of all main effects and two-way interactions ensure that even if two-way interactions are significant, they are independent of main effects, minimising bias if interactions are omitted [57]. In summary, we would recommend fractional factorial design using a D-efficient measure that allows estimation of main effects with limited interactions considering theory, intuition, feasibility in terms of sample size and survey design [58].

### Designing choice sets

In the context of screening, an opt out is likely to be important because the reality is that many people do opt out of screening resulting in health screening uptake remains low in many countries [13, 14]. The inclusion of an opt out option in a DCE or choose neither option may have an effect on choice behaviour [3, 59]. Thus, it is recommended to allow respondents to opt out or choose neither option that can reveal the reality of health screening uptake. More than half of reviewed studies offered an opt-out option. Since offering opt-out options, a dual response design is strongly recommended to increase data quality [59]. In a dual response design, participants first make a forced choice and then participants are asked if they would like to opt-out if given the choice [60]. This might reduce the risk that a direct introduction of an opt-out results in large numbers of respondents avoiding to seriously weigh the different levels of attributes [59].

### Analysis methods

The current review found considerable variation in methods of econometric analyses which raises concern about the validity of the results. The ISPOR guidelines and more importantly theoretical underpinning suggests conditional logit as one of the most common analysis methods [61]. Other models such as scale multinomial logit, generalised multinomial logit and mixed logit have also been recommended. The conditional logit model was first suggested by McFadden [62] proposing random utility model which describes the choice among alternatives due to the characteristics of attribute levels defining those alternatives [61]. Multinomial logit is similar to conditional logit but the choices with multinomial logit are more reflective of the characteristics of the respondents [61, 63]. Of the 27 reviewed studies only ten had used one of the recommended analysis methods and three had used conditional logit. Others had not justified the use of different analysis models. Our recommendation is to follow the ISPOR guidelines on best practices [61, 64]. For evaluating the goodness-of-fit of the model, pseudo *R*^2^ measures and AIC or BIC are ideal techniques. However, both have strengths and limitations. AIC and BIC focus on minimizing underestimation rather than checking the adequacy of the model to explain responses in the data as done in the pseudo *R*^2^ measures. Our review findings were consistent with these guidelines and we would recommend using goodness of fit measures such as likelihood function measures with Pseudo *R*^2^, BIC, and AIC to compare and/or select a model.

### Sample size

The sample size for DCE study depends on the chosen statistical model, significance level, statistical power level, the experimental design, and likely parameter estimates [65, 66]. Consequently, the reviewed studies used a wide range of sample sizes as well as a variety of sample size calculation methods. Initially, in the healthcare sector, the number of eligible public informants and healthcare providers is generally limited. Consequently, minimum and adequate sample size is needed to have statistical power to detect a difference in preferences when this difference is sufficiently large [65]. Studies that were published in the last 2 years [47, 51, 54] applied the minimum sample size for health care related DCE studies proposed by de Bekker-Grob, Donkers [65]. Compared to other methods such as parametric approach, rule of thumb, and referring studies, this approach was better suited to determine the minimum required sample size for hypothesis testing for coefficients based on DCEs, and can be extended to functions of parameters [65]. The parametric approach can only be used if prior parameter estimates are available and are not equal to zero [66]. The rule of thumb methods are not intended to be strictly accurate or reliable [65]. The number of participants approached varied from very small (*n* = 46) to large (*n* = 4000). However, the average size was approximately 1000 when the general population or clinicians were surveyed.

### Survey method

Compared with face-to-face interview and paper-based methods, online surveys are cheaper and save time [67]. This review found that response rates were high when studies used face-to-face interview methods to collect data, while response rates varied in studies where DCEs were administered to an online survey panel. We recommend using face to face data collection methods to improve validity and failing that, a known contact list to collect data from clinicians and patients. When DCE data collection involve general population, the convenient method is to use a valid online panel.

### Strengths and limitations

This study focuses on the technical aspects of DCE studies in relation to their use in community health screening programs. The number of DCE studies is increasing over time, and our work can serve both as a guide to the details of conducting a DCE and as a practical aid to future research. We have highlighted several areas of current practice that can be improved, particularly greater use of sample size calculations, increased use of qualitative methods, better reporting of how choice sets are generated, and explanation of chosen experimental design and analysis method.

A limitation of this scoping review was the reliance on what was reported in the studies published in English language between Jan 2011 and Mar 2021. Additionally, abstracts based on a conference presentation were excluded.

## Conclusion

Discrete Choice Experiments of community health screening programmes vary substantially with respect to developing choice sets and generating experimental design. These variations limit the overall conclusions and generalisability of the results in policy contexts. Nevertheless, based on information obtained from the studies included in this review, our findings provide a series of recommendations for future DCE studies related to community-based health screening programmes.

## Supplementary Information

**Additional file 1.**

**Additional file 2.**

**Additional file 3.**

## Data Availability

All data generated or analysed during this study are included in this published article and its supplementary information files.
