# Supplementary material for: Methodology to derive preference for health screening programmes using discrete choice experiments: a scoping review
Source: BMC Health Serv Res. 2022 Aug 24;22:1079. doi: 10.1186/s12913-022-08464-7 (PMC9400308; doi:10.1186/s12913-022-08464-7)
Supplement: Supplementary file 1 — Additional file 1. [file 12913_2022_8464_MOESM1_ESM.docx]

**Appendix 1**

**Preferred Reporting Items for Systematic reviews and Meta-Analyses extension for Scoping Reviews (PRISMA-ScR) Checklist**

| **SECTION** | **ITEM** | **PRISMA-ScR CHECKLIST ITEM** | **REPORTED ON PAGE #** |
| --- | --- | --- | --- |
| **TITLE** | | | |
| Title | 1 | Identify the report as a scoping review. | Page 1 |
| **ABSTRACT** | | | |
| Structured summary | 2 | Provide a structured summary that includes (as applicable): background, objectives, eligibility criteria, sources of evidence, charting methods, results, and conclusions that relate to the review questions and objectives. | Page 2 |
| **INTRODUCTION** | | | |
| Rationale | 3 | Describe the rationale for the review in the context of what is already known. Explain why the review questions/objectives lend themselves to a scoping review approach. | Pages 4-5 |
| Objectives | 4 | Provide an explicit statement of the questions and objectives being addressed with reference to their key elements (e.g., population or participants, concepts, and context) or other relevant key elements used to conceptualize the review questions and/or objectives. | Page 5 |
| **METHODS** | | | |
| Protocol and registration | 5 | Indicate whether a review protocol exists; state if and where it can be accessed (e.g., a Web address); and if available, provide registration information, including the registration number. | Appendix 2 |
| Eligibility criteria | 6 | Specify characteristics of the sources of evidence used as eligibility criteria (e.g., years considered, language, and publication status), and provide a rationale. | Page 6;  Appendix 2 |
| Information sources* | 7 | Describe all information sources in the search (e.g., databases with dates of coverage and contact with authors to identify additional sources), as well as the date the most recent search was executed. | Page 6-7;  Appendix 2 |
| Search | 8 | Present the full electronic search strategy for at least 1 database, including any limits used, such that it could be repeated. | Page 6-7;  Appendix 2 |
| Selection of sources of evidence† | 9 | State the process for selecting sources of evidence (i.e., screening and eligibility) included in the scoping review. | Page 6-7;  Appendix 2 |
| Data charting process‡ | 10 | Describe the methods of charting data from the included sources of evidence (e.g., calibrated forms or forms that have been tested by the team before their use, and whether data charting was done independently or in duplicate) and any processes for obtaining and confirming data from investigators. | Page 7;  Appendix 2 |
| Data items | 11 | List and define all variables for which data were sought and any assumptions and simplifications made. | Page 7-8;  Appendix 2 |
| Critical appraisal of individual sources of evidence§ | 12 | If done, provide a rationale for conducting a critical appraisal of included sources of evidence; describe the methods used and how this information was used in any data synthesis (if appropriate). | Page 8; |
| Synthesis of results | 13 | Describe the methods of handling and summarizing the data that were charted. | Page 7-8 |
| **RESULTS** | | | |
| Selection of sources of evidence | 14 | Give numbers of sources of evidence screened, assessed for eligibility, and included in the review, with reasons for exclusions at each stage, ideally using a flow diagram. | Page 8-9 |
| Characteristics of sources of evidence | 15 | For each source of evidence, present characteristics for which data were charted and provide the citations. | Page 10-19  Appendix 3 |
| Critical appraisal within sources of evidence | 16 | If done, present data on critical appraisal of included sources of evidence (see item 12). | Page 10;  Appendix 4 |
| Results of individual sources of evidence | 17 | For each included source of evidence, present the relevant data that were charted that relate to the review questions and objectives. | Appendix 3 |
| Synthesis of results | 18 | Summarize and/or present the charting results as they relate to the review questions and objectives. | Page 10-19 |
| **DISCUSSION** | | | |
| Summary of evidence | 19 | Summarize the main results (including an overview of concepts, themes, and types of evidence available), link to the review questions and objectives, and consider the relevance to key groups. | Page 20-24 |
| Limitations | 20 | Discuss the limitations of the scoping review process. | Page 24 |
| Conclusions | 21 | Provide a general interpretation of the results with respect to the review questions and objectives, as well as potential implications and/or next steps. | Page 24-25 |
| **FUNDING** | | | |
| Funding | 22 | Describe sources of funding for the included sources of evidence, as well as sources of funding for the scoping review. Describe the role of the funders of the scoping review. | Page 25 |

JBI = Joanna Briggs Institute; PRISMA-ScR = Preferred Reporting Items for Systematic reviews and Meta-Analyses extension for Scoping Reviews.

* Where *sources of evidence* (see second footnote) are compiled from, such as bibliographic databases, social media platforms, and Web sites.

† A more inclusive/heterogeneous term used to account for the different types of evidence or data sources (e.g., quantitative and/or qualitative research, expert opinion, and policy documents) that may be eligible in a scoping review as opposed to only studies. This is not to be confused with *information sources* (see first footnote).

‡ The frameworks by Arksey and O’Malley (6) and Levac and colleagues (7) and the JBI guidance (4, 5) refer to the process of data extraction in a scoping review as data charting*.*

§ The process of systematically examining research evidence to assess its validity, results, and relevance before using it to inform a decision. This term is used for items 12 and 19 instead of "risk of bias" (which is more applicable to systematic reviews of interventions) to include and acknowledge the various sources of evidence that may be used in a scoping review (e.g., quantitative and/or qualitative research, expert opinion, and policy document).

*From:* Tricco AC, Lillie E, Zarin W, O'Brien KK, Colquhoun H, Levac D, et al. PRISMA Extension for Scoping Reviews (PRISMAScR): Checklist and Explanation. Ann Intern Med. 2018;169:467–473. doi: 10.7326/M18-0850.

# **Appendix 2**

**Review protocol**

**Title:** Methodology to derive preference for health screening programmes using Discrete Choice Experiments: A scoping review

**Review question:** To summarise and critically appraise the published evidence relating to the use of discrete choice experiments to elicit health preferences where the intervention is a screening tool and/or model of care, focusing specifically on the purpose of the studies, the types of attributes and levels included, and key methodical features of the application of discrete choice methods.

**Searches:**

Reviews will be conducted according to PRISMA guidelines [1] and include searches of electronic databases plus searching official websites and reference lists. There will be original research using a discrete choice experiment to elicit health preferences where the intervention is a screening tool and/or model of care.

***Data sources:*** Three electronic databases - Medline, PubMed, EMBASE and the National Health Service Economic Evaluation Databases will be searched with the assistance of librarians. The search will cover the period from Jan 2000 to Feb 2021. English language only.

**Search terms:**

| **SPICE elements** | **Search terms assigned** | **Reason** |
| --- | --- | --- |
| Setting - where? | Not term assigned | The interests of the review include all contexts |
| Population - whom? | 1. **Consumers:** “patient* preference” OR “consumer* preference” OR “public preference” OR “individual* preference” OR “person* preference” OR “citizen* preference” OR “resident* preference” OR “adult* preference” OR “people* preference”, OR “child* preference”, “kid* preference” OR “adolescen* preference” OR “teenager* preference” | To include the information to consumers, providers as well as policymakers. |
|  | 1. **Providers:** “health* worker* preference” OR “health* personnel* preference” OR “health* professional* preference” OR “hospital staff* preference” OR “doctor* preference” OR “physician* preference” OR “clinic* preference” OR “medic* preference” OR “nurse* preference” OR “midwife* preference” OR “pharmac* preference*” OR “health practition* preference” OR ”health care provider* preference” |  |
|  | 1. **Policymakers:** “stakeholder* preference” OR “policymaker* preference” OR “decision-maker* preference” OR “legislator* preference” OR “lawmaker* preference” OR “official* preference” OR |  |
| Intervention – What? | “discrete choice*” OR “choice experiment*” OR “stated preference*” OR “discrete choice model*” OR “part‐worth utilities” OR “functional measurement” OR “paired comparisons” OR “pairwise choice*” OR “conjoint analysis*” OR “conjoint measurement*” OR “conjoint stud*” OR “conjoint choice experiment*” OR “dce” | The intervention of interests is the discrete choice experiments |
| Comparison – compared to what? | Not term assigned | Not interested in comparing different methodologies or programs |
| Evaluation – with what result? | “Screening” OR “surveillance” OR “intervention” OR “health exanimation” OR “health assessment” OR “health screen test” OR “health investigation” OR “explor*” AND “commun*” OR “public*” OR “social” OR “municipal” OR “local*” OR “national*” | The outcomes of interest are the choices for community health screening programs |

***Search strategy:***

|  | **Search terms** | **Numbers** |
| --- | --- | --- |
| 1. **PUBMED** | | |
| 1. | preference* OR choice* OR attitude* OR value* | 3,116,010 |
| 2. | patient* OR consumer* OR population* OR resident* OR adult* OR child* OR individual* OR teen* OR citizen* OR public | 14,084,520 |
| 3. | "health* worker*" OR "health* personnel*" OR "health* professional*" OR "hospital staff*" OR doctor* OR physician* OR nurse* OR midwife* OR "health* practitioner*" OR pharmacist* OR clinician* | 1,612,803 |
| 4. | "Health Personnel"[Mesh] | 532,099 |
| 5. | stakeholder* OR policymaker* OR "decision maker*" OR legislator* OR lawmaker* OR official* | 123,030 |
| 6. | #2 OR #3 OR #4 OR #5 | 14,691,302 |
| 7. | "discrete choice*" OR "choice experiment*" OR "stated preference*" OR "discrete choice model*" OR "part‐worth utilities" OR "functional measurement" OR "paired comparisons" OR "pairwise choice*" OR "conjoint analysis*" OR "conjoint measurement*" OR "conjoint stud*" OR "conjoint choice experiment*" | 6,235 |
| 8. | "screening" OR "surveillance" OR "intervention" OR "health exanimation" OR "health assessment" OR "health screen test" OR "health investigation" OR explor* | 2,446,387 |
| 9. | ("Early Detection of Cancer"[Mesh]) OR "Mass Screening"[Mesh] | 152,582 |
| 10. | #8 OR #9 | 2,460,343 |
| 11. | #1 AND #6 AND #7 AND #10 | 952 |
| 12. | Limited 2000-2021 and only English | 940 |
| 1. **EMBASE** | | |
| 1. | preference* OR choice* OR attitude* OR 'value'/exp OR value | 2,931,317 |
| 2. | patient* OR consumer* OR population* OR resident* OR adult* OR child* OR individual* OR teen* OR citizen* OR public | 18,991,358 |
| 3. | 'health* worker*' OR 'health* personnel*' OR 'health* professional*' OR 'hospital staff*' OR doctor* OR physician* OR nurse* OR midwife* OR 'health* practitioner*' OR pharmacist* OR clinician* | 2,298,698 |
| 4. | 'health care personnel'/exp | 1,671,058 |
| 5. | stakeholder* OR policymaker* OR 'decision maker*' OR legislator* OR lawmaker* OR official* | 381,344 |
| 6. | #2 OR #3 OR #4 OR #5 | 19,876,130 |
| 7. | 'discrete choice*' OR 'choice experiment*' OR 'stated preference*' OR 'discrete choice model*' OR 'part‐worth utilities' OR 'functional measurement' OR 'paired comparisons' OR 'pairwise choice*' OR 'conjoint analysis*' OR 'conjoint measurement*' OR 'conjoint stud*' OR 'conjoint choice experiment*' | 8,119 |
| 8. | 'screening' OR 'surveillance' OR 'intervention' OR 'health exanimation' OR 'health assessment' OR 'health screen test' OR 'health investigation' OR explor* | 3,588,923 |
| 9. | #1 AND #6 AND #7 AND #8 | 1,326 |
| 10. | Limited 2000-2021 | 1,315 |
| 1. **MEDLINE** | | |
|  | ( preference* OR choice* OR attitude* OR value* ) AND ( patient* OR consumer* OR population* OR resident* OR adult* OR child* OR individual* OR teen* OR citizen* OR public OR"health* worker*" OR "health* personnel*" OR "health* professional*" OR "hospital staff*" OR doctor* OR physician* OR nurse* OR midwife* OR "health* practitioner*" OR pharmacist* OR clinician* OR stakeholder* OR policymaker* OR "decision maker*" OR legislator* OR lawmaker* OR official* ) AND ( "discrete choice*" OR "choice experiment*" OR "stated preference*" OR "discrete choice model*" OR "part‐worth utilities" OR "functional measurement" OR "paired comparisons" OR "pairwise choice*" OR "conjoint analysis*" OR "conjoint measurement*" OR "conjoint stud*" OR "conjoint choice experiment*" ) AND ( "screening" OR "surveillance" OR "intervention" OR "health exanimation" OR "health assessment" OR "health screen test" OR "health investigation" OR explor* )  **Limiters** - Date of Publication: 20000101-20211231  **Expanders** - Apply equivalent subjects  **Narrow by Language:**- english  **Search modes** - Boolean/Phrase | 929 |
| 1. **National Health Service Economic Evaluation Databases up to 2015** | | |
|  | ((discrete choice* OR choice experiment* OR stated preference* OR discrete choice model* OR part‐worth utilities OR functional measurement OR paired comparisons OR pairwise choice* OR conjoint analysis* OR conjoint measurement* OR conjoint stud* OR conjoint choice experiment*)) and ((Economic evaluation and Bibliographic) OR (Economic evaluation and Abstract:) OR Project record OR Full publication record) IN NHSEED, HTA FROM 2000 TO 2021 WHERE LPD FROM 01/01/2000 TO 03/03/2021 | 13 |

***Additional searching:***

- Official websites
- Reference list review of any article pulled for possible inclusion
- Contact with study authors

***Question of interest*:** What is the current evidence for using a discrete choice experiment to elicit health preferences where the intervention is a screening tool and/or model of care?

***Population*:** Patients or general population of any age or sex who have been involved in a screening tool and/or model of care, health care providers, and policy makers.

***Intervention(s), exposure(s):***

The use of discrete choice experiments only to elicit health preferences where the intervention is a screening tool and/or model of care.

***Comparator(s)/control:*** Not applicable

***Study designs of interest*:** Studies assessing a screening tool and/or model of care using discrete choice experiments only.

***Inclusion criteria:***

1. Studies will be included if they applied a discrete choice experiment method to elicit consumer preferences towards intervention (a screening tool/model of care)
2. Research articles published in English language
3. Research limited to human studies
4. Full publication or manuscript available for review

***Exclusion criteria:***

1. Not published in English
2. Editorials, unpublished grey literature, guidelines, conference proceedings, methodology and protocol papers and literature reviews.
3. Articles initially excluded if they are duplicates or if the title/abstract clearly demonstrates that the study objective and outcome are not the focus of the review.
4. Prenatal screening, newborn screening, sexual transmitted infection screening as well as genetical testing

***Main outcome(s)***

To identify the key methods used to elicit health preferences using discrete choice experiments.

To present discuss the attributes used in discrete choice experiments concerning a screening tool/model of care, and discuss the DCE design and analysis methods.

***Additional outcome(s)***

To investigate preferences for a screening tool/model of care, including measures of willingness to pay, marginal rates of substitution between attributes, and estimates of demand.

***Measures of effect***

Not applicable

***Data extraction***

The initial search will be performed by two reviewers using pre-determined search terms and strategies from chosen databases. All identified papers will be imported into EndNote and will be screened in accordance with PRISMA ScR guidelines [2]. Review will be carried out using Rayyan Software [3].

After removal of duplicates, the titles and abstracts will be screened for relevance and eligibility criteria. The two reviewers will then extract the data from the studies selected for inclusion using a predesigned extraction form. The data extraction sheet will be first pilot tested on five studies and then will be revised accordingly to include:

*Identification of study:*

1. Record the first authors’ last name, initials
2. Record the journal name
3. Record the year of publication
4. Record the volume and page numbers

*Characteristics of study:*

1. Study setting
2. Time horizon/Study period
3. Research question
4. Participants type
5. Sample size
6. Type of intervention (screening tool or model of care)
7. Study objective
8. Survey mode and response rate
9. Study design
   1. Design type
   2. Attributes and attribute levels
   3. Design plan
   4. Design software
10. Study analysis
    1. Type of modelling
    2. Analysis software
    3. Relative attribute impact analysis
    4. Validity analysis
       1. Content validity
       2. External validity
11. Interpretation and policy analysis
    1. Probability analysis
    2. Marginal rates of substitution
    3. Welfare measures to value health and healthcare

***Risk of bias (quality) assessment***

The full text of all included articles will be assessed for reporting quality by two independent reviewers by using a list of criteria for studies applied discrete choice experiments used in the review study conducted by Mandeville et al., [4]. Any discrepancies over reporting quality assessment between the two reviewers will be resolved by discussion with a third reviewer.

***Strategy for data synthesis***

After screening the title abstract and full text, data will be extracted from relevant articles and will be summarised in tables. The data fields will be; research question, setting and location, study design, study analysis and validity analysis.

The quality of the included studies will be assessed using a list of 13 criteria, which covers all four key stages of a discrete choice experiment, used in Mandeville et al., [4]. Each item in the checklist were scored as having ‘met the criteria in full’ (‘1’) and ‘partially met or did not meet the criteria’ (‘0’). Overall compliance with the checklist was calculated as the proportion of the checklist criteria addressed by the study.

***Analysis of subgroups or subsets***

None

**Refences for Appendix 2**

1. Page M. J., Moher D., Bossuyt P., Boutron I., Hoffmann T., Mulrow C. et al. PRISMA 2020 explanation and elaboration: updated guidance and exemplars for reporting systematic reviews. 2020, to be published.

2. Peters M. D., Godfrey C. M., McInerney P., Munn Z., Tricco A. C., Khalil H. et al., "Scoping reviews," vol. 18, JBI Manual for Evidence Synthesis, Aromataris E and M. Z, Eds., 2020, pp. 2119-2126. [Online]. Available: https://doi.org/10.46658/JBIMES-20-12.

3. Ouzzani M., Hammady H., Fedorowicz Z., and Elmagarmid A. Rayyan-a web and mobile app for systematic reviews. Systematic Reviews, 5, 210. 2016, to be published.

4. Mandeville K. L., Lagarde M., and Hanson K. The use of discrete choice experiments to inform health workforce policy: a systematic review. *BMC Health Services Research*, 2014/09/01 2014, 14, 367.

# **Appendix 5**

**Table 1 List of items for developing attributes for the choice sets in a DCE of community screening programme.**

|  | Action required by you personally to arrange test |
| --- | --- |
|  | Availability follow up test |
|  | Changes to organ(s) such as breast |
|  | Cognitive function |
|  | Co-morbidity |
|  | Cost your test to a national health system |
|  | Discomfort after procedure |
|  | Discomfort during procedure |
|  | Effectiveness |
|  | False negative |
|  | False positive |
|  | Financial incentive |
|  | Frequency of procedure |
|  | Functional status |
|  | Generation of knowledge and effectiveness |
|  | Healthcare provider recommendation |
|  | Identification of population at risk |
|  | Listing of patients |
|  | Location |
|  | Maximization of follow up and treatment |
|  | Maximization of uptake |
|  | Method of screening |
|  | Mortality |
|  | Nature of procedure |
|  | Number of procedures |
|  | Nurse assistance prior to appointment |
|  | Operation of the program |
|  | Out of pocket cost for follow up care |
|  | Out of pocket cost for screening procedure |
|  | Overdiagnosis |
|  | Patient's age |
|  | Practitioner's sex |
|  | Preparation for procedure |
|  | Procedure information given prior to test |
|  | Procedure options |
|  | Procedure reminder |
|  | Procedure sensitivity |
|  | Procedure specificity |
|  | Reduction in mortality |
|  | Risk of later regret |
|  | Risk of overtreatment |
|  | Risk of unnecessary biopsy |
|  | Risk of unnecessary treatment |
|  | Scientific evidence |
|  | Screening duration |
|  | Screening interval |
|  | Sexual changes after procedure |
|  | Side effects of procedure |
|  | Staff assistance |
|  | Stakeholder's acting |
|  | Survival rate |
|  | Target population |
|  | Training for healthcare providers |
|  | Travel time |
|  | Waiting time for the screening procedure |
|  | Waiting time for the follow up test |
|  | Waiting time for the results |
|  | Who reviews the results |
