# Supplementary material for: Methodology to derive preference for health screening programmes using discrete choice experiments: a scoping review
Source: BMC Health Serv Res. 2022 Aug 24;22:1079. doi: 10.1186/s12913-022-08464-7 (PMC9400308; doi:10.1186/s12913-022-08464-7)
Supplement: Supplementary file 3 — Additional file 3. [file 12913_2022_8464_MOESM3_ESM.docx]

Overview table

| **Author (publication date)** | **Country** | **Type of population** | **Sample size** | **Overall aim/objective** | **Main outcome** |
| --- | --- | --- | --- | --- | --- |
| Walters et al (2011) | UK | Healthcare provider | 488 | Health professionals' preferences for treatment or screening options for patients | Relative risk |
| De Bekker-Grob et al (2013) | Netherlands | General population | 1000 | Investigating trade-offs health outcomes and patient or consumer experience factors | Utility score, WTP |
| Benning et al. (2014)  VALUE IN HEALTH | Netherlands | General population | 1277 | Consumer preference | Choice shares |
| Benning et al. (2014)  ACTA ONCOLOGA | Netherlands | General population | 1575 | Consumer preference | Utility score |
| Pignone et al (2014) | US | General population | 175 | Consumer preference | Utility score |
| Howard et al (2015) | Australia | General population | 793 | Investigating trade-offs health outcomes and patient or consumer experience factors | Utility score, WTA, WTP |
| Kitchener et al (2016) | UK | General population | 4000 | Consumer preference | Utility score |
| Martens et al (2015) | US | General population | 46 | Consumer preference | Utility score |
| Sicsic et al (2016) | France | Healthcare provider | 685 | Health professionals' preferences for treatment or screening options for patients | Utility score |
| Spinks et al (2016) | Australia | General population | 50 | Consumer preference | Utility score, marginal WTP (MWTP) |
| Papin-Lefebvre et al (2017) | France | Healthcare provider | 2114 | Health professionals' preferences for treatment or screening options for patients | Utility score |
| Mansfield et al (2018) | US | General population | 3263 | Consumer preference | Utility score, WTP |
| Sicsic et al (2018) | France | General population | 1102 | Investigating trade-offs health outcomes and patient or consumer experience factors | Utility score, WTA |
| Snoswell et al (2018) | Australia | General population | Not reported | Consumer preference | Utility score, marginal WTP |
| Byrne et al (2019) | US | General population | 525 | Consumer preference | Relative importance and ranking, part-worth utility score |
| Hansen et al (2019) | UK | General population | 1231 | Consumer preference | Utility score, WTA |
| Li et al (2019) | China | General population | 420 | Consumer preference | Utility score, WTP, uptake rate |
| Mandrik et al (2019) | Belarus | General population | 490 | Consumer preference | Utility score |
| Priaulx et al (2019) | Europe | Healthcare provider and stakeholders | 66 | Health professionals' preferences for treatment or screening options for patients | Relative importance and ranking, utility score |
| Bilger et at (2020) | Singapore | General population | 401 | Consumer preference | Utility score, uptake rate |
| Charvin et al (2020) | France | General population | 2703 | Consumer preference | Utility scores, WTP |
| De Bekker-Grob et al 2020 | Netherlands | General population | 485 | Consumer preference | Utility score |
| Hendrix et al (2020) | US | Healthcare provider | 350 | Health professionals' preferences for treatment or screening options for patients | Relative importance and ranking |
| Norman et al (2020) | Australia | General population | 521 | Consumer preference | Utlity score, WTP |
| Peters et al (2020)  VALUE IN HEALTH | Netherlands | General population | 1000 | Consumer preference | Utility score, WTP, uptake rate |
| Peters et al (2020)  CLINICAL AND TRANSLATIONAL GASTROENTROLOGY | Netherlands | General population | 1500 | Consumer preference | Part-worth utility score, maximum acceptable risk, uptake rate |
| Raginel et al (2020) | France | Healthcare provider | 2684 | Health professionals' preferences for treatment or screening options for patients | Utility score |

*WTP = willingness to pay, WTA = willingness to accept.

** Where author name and year was same, journal name was used.
